# Regional variation in health care substitution for intrauterine device insertion: a retrospective cohort study

**DOI:** 10.1186/s12875-024-02546-7

**Published:** 2024-08-10

**Authors:** Maarten D. Vink, France R. Portrait, Tim van Wezep, Xander Koolman, Ben W. Mol, Eric J. van der Hijden

**Affiliations:** 1 Department of Health Economics, School of Business and Economics &, Talma Institute, Vrije Universiteit, De Boelelaan 1085, 1081 HV Amsterdam, The Netherlands; 2 Department of Obstetrics and Gynaecology, Meander Medical Center, Maatweg 3, 3813 TZ Amersfoort, The Netherlands; 3 Vektis, Sparrenheuvel 18, 3708 JE Zeist, The Netherlands; 4 Department of Obstetrics and Gynaecology, Monash University, 246 Clayton Road, Clayton , Victoria, 3168 Australia; 5 Zilveren Kruis Health Insurance, Handelsweg 2, 3707 NH Zeist, The Netherlands

**Keywords:** Care substitution, IUD insertion, Medical practice variation, Contraception, Cohort study

## Abstract

**Background:**

Rising health care costs are a major concern in most Western countries. The substitution of healthcare stands as a strategic approach aimed at mitigating costs while offering medical services in proximity to patients’ residences. An illustrative instance involves the migration of outpatient hospital care to primary care settings. Notably, the insertion of intrauterine devices (IUDs) can be safely executed within primary care contexts. In order to establish a pragmatic objective for the rate of IUD substitution, we conducted an evaluation of regional disparities in healthcare substitution pertaining to the insertion of intrauterine devices. Furthermore, we investigated disparities in the follow-up ultrasound and reinsertion of IUDs between primary and secondary healthcare environments.

**Methods:**

All women who underwent IUD insertion in Dutch primary care (by general practitioners and midwives) and secondary care (by hospital physicians) between January 1, 2016, and December 31, 2020 were included. The main outcome measures were the case-mix adjusted IUD insertion rates at the regional level by care setting and the proportions requiring follow-up ultrasound and IUD reinsertion within three months.

**Results:**

Of the 840,766 IUD placements, 74% were inserted in primary care and 26% in secondary care. The proportion inserted in primary care increased from 70% in 2016 to 77% in 2020. The observed substitution rate ranged from 58 to 82% between regions. Compared with health care professionals in primary care, those in secondary care performed more ultrasounds to verify IUD placement (23% vs. 3%; p-value < 0.01) and more IUD reinsertions within three months (6% vs. 2%; p-value < 0.01).

**Conclusions:**

IUDs are increasingly being inserted in Dutch primary care, with peak regional IUD insertion care substitution rates at ≥ 80%. IUD insertion care substitution to primary care appears to be associated with significantly fewer women having follow-up ultrasound or IUD reinsertion within three months.

**Supplementary Information:**

The online version contains supplementary material available at 10.1186/s12875-024-02546-7.

## Background

High health care costs are a major concern in most Western countries [1], with costs only expected to rise further. In Europe, overall health care spending is likely to increase by 0.8% annually until 2050 if policy, health care organization and care delivery remain unchanged [2], which could limit the resources available to other public services if overall spending does not increase. Despite the complexity of this problem, several policy strategies have been proposed to contain health care costs, primarily by reducing the cost of hospital care though price and volume controls or through policies that force health care providers to meet strict budgetary targets [3]. An alternative strategy is to focus on health care substitution.

Health care substitution is defined as “the continual regrouping of resources across and within care settings to exploit the best, and least costly, solution in the face of changing needs and demands” [4]. An example of care substitution across settings is the transfer of outpatient hospital care to primary care. These “primary care plus” services can be provided by either the general practitioner (GP) or a member of hospital staff working in primary care [5]. By contrast, an example of health care substitution within care settings is the transfer of tasks from physicians to other health care professionals, called professional role substitution. Both forms of care substitution have been shown to save resources without compromising the quality of health care [5, 6]. On this basis, the Dutch government launched a campaign in 2018 called “the right care at the right place” to reduce the number of hospital referrals made by GPs. In the Netherlands, GPs serve as gatekeepers to secondary care, with patients required to consult with them initially for most health complaints. Although referral to a medical specialist should only occur if their health complaint cannot be resolved in primary care, the reality is that referrals also occur due to strong patient preference and high GP workloads. Reducing the number of unnecessary referrals could not only save money but also increase patient satisfaction by providing care closer to home. An evaluation of the campaign in 2021 showed that this had improved cooperation between health care organizations, but it failed to assess the change in referral rate from primary to secondary care [7].

Intrauterine devices (IUDs) are used as methods of contraception and treatments for heavy menstrual bleeding, and their insertion represents a clear example of where care substitution can be achieved with relative ease. IUDs can be inserted safely by GPs, gynaecologists, and from 2017, midwives [8]. Midwives in the Netherlands work as independent practitioners in primary care or as clinical midwives in a hospital setting. Since 2017, midwives are allowed to insert IUDs in primary care within their own practices. Women can make an appointment for IUD insertion directly, without a referral by the GP, regardless of whether they have ever been pregnant.

The cost of IUD insertion in the Netherlands comprises two components: material costs and insertion costs. The material cost ranges from approximately 60 euros for a copper IUD to approximately 150 euros for a hormonal IUD [9]. The insertion cost in primary care is approximately 75 euros, while insertion by a gynaecologist in a hospital costs approximately 250 euros [10]. The reimbursement for IUDs is contingent upon the indication for placement, whether for contraception or for the management of bleeding disorders. For contraceptive purposes, the material costs of IUDs are not covered by basic insurance unless the woman is under 21 years of age. When the insertion is conducted in primary care settings, the procedure is typically reimbursed by basic insurance, resulting in the woman only being responsible for the material costs. Conversely, if the insertion occurs in secondary care, the costs associated with the procedure itself are not covered by basic insurance, thereby requiring the woman to cover both the material and insertion costs. In cases where the IUD is indicated for bleeding disorders, the material costs are not covered when the woman purchases the device from the pharmacy. However, if the IUD is placed in a hospital setting by a gynaecologist, the costs are reimbursed by the insurer only after the deductible of 385 euros has been exceeded. The placement for this indication is reimbursed by the insurer in both primary and secondary care settings, although the latter also involves the mandatory deductible of 385 euros. Additionally, if a woman has supplementary insurance, she may be eligible for broader coverage for both the material and insertion costs, for either indication of contraception or bleeding disorders [11].

Consequently, shifting IUD insertions from secondary to primary care results in a cost reduction. Depending on the situation (indication of placement and whether a woman has supplementary insurance), the benefit of cost reduction through care substitution may accrue to the individual woman or the health insurer. However, in all cases, it is important to shift care from secondary to primary care to reduce healthcare costs. Although reports show that regional variation exists in IUD insertion rates per capita among GPs [12], these reports lack data about regional variation in care substitution from secondary to primary care for IUD insertion. To better identify the potential for improvement through the substitution of IUD insertion care, it is necessary to better map the practice variation in this substitution. This study therefore aims to investigate regional variations in IUD substitution practices, with the goal of identifying a feasible target for increasing the IUD substitution rate. By doing so, we aim to decrease unnecessary hospital referrals for IUD insertion, which are linked to higher costs. Additionally, we seek to evaluate the potential effect of increased substitution on differences in follow-up between primary and secondary care.

## Methods

### Study design and participants

We performed a retrospective, observational cohort study using claims data from Vektis, the executive agency of the umbrella organization for all health insurers in the Netherlands (*Zorgverzekeraars Nederland*), with follow-up data collected for 3 months. The study population comprised all Dutch women who had an IUD inserted between January 1st, 2016, and December 31st, 2020, with an age between 15 and 55 years at the time of insertion.

The compliance officers of Vektis approved the study, for which the use of retrospective data collection and anonymous analysis exempted the need for institutional review board approval.

### Datasets

Vektis receives claims data from all insurance companies for care provided in primary and secondary settings in the Netherlands. For IUD insertion, their dataset included who performed the insertion and when, together with any subsequent treatments during follow-up (e.g., ultrasound or new IUD insertion). In the Netherlands, contraception guidelines for GPs and gynaecologists state that follow-up ultrasound is not useful and should not routinely be performed unless IUD insertion was difficult and malposition is suspected [13]. GPs also typically refer patients to a hospital when they require an ultrasound. Therefore, follow-up ultrasounds performed in a hospital, excluding those on the day of insertion for the IUDs inserted in hospital setting, were considered follow-up ultrasounds to locate the IUD. Additionally, we considered the first insertion unsuccessful if a new IUD was inserted within 3 months.

All participants were allocated to a specific COROP region (*Coördinatiecommissie Regionaal Onderzoeksprogramma*; townships belonging to an arbitrary geographic region) using their postal code at the time of IUD insertion. This regional division is commonly used in the Netherlands for research into practice variation. Finally, to perform case-mix correction by demographic and socioeconomic characteristics, the claims data from Vektis were enriched with data by postal code from Statistics Netherlands for 2018 [14].

### Outcome measures

We set one primary and four secondary outcome measures, which we assessed both nationally and by COROP region. The primary outcome was the percentage of IUDs inserted in primary care, calculated by dividing the number of IUDs inserted by GPs and midwives by the total number of inserted IUDs. We expressed the number of IUDs per type of healthcare professional per 100,000 person-years per COROP region. This approach not only allows calculation of the fraction of IUD insertions by type of healthcare professional and region but also describes the population-level uptake of IUDs. The secondary outcomes were the percentage of women who underwent follow-up ultrasound within 3 months after IUD insertion and the percentage of women who underwent IUD reinsertion within 3 months, stratified by primary and secondary care.

### Statistical analyses

We used SAS version 9.4 (SAS Institute Inc., Cary, NC, USA) for data storage and analysis. Descriptive statistics are reported for each outcome measure and baseline characteristic, including the crude number of IUDs inserted per 100,000 person years by COROP region (stratified by health care professional). The primary and secondary outcomes were adjusted for case-mix variables, using five multilevel random effects logit models, because the independence of measurements within each COROP region could not be assumed. For the primary outcome, we also estimated the fully adjusted models by calendar year. For the secondary outcomes, Student’s T test was used to compare means between two groups. The definitions and health care activity codes used to construct the models are detailed in Appendix A.

Literature shows that IUD placement can be more difficult if women have not have given birth [15], if they are obese, which correlates with low socioeconomic status in high-income countries [16], and if they are from certain ethnic groups prone to uterine myomas [17]. Therefore, we performed case-mix correction for age (a proxy for childbearing), household income (a proxy for socioeconomic status), and percentage of non-Western immigrants (a proxy for ethnicity).

COROP-specific random parameter estimates were retrieved for each model to calculate the adjusted rate per COROP region, allowing assessment of the extent to which single regions produced rates consistently above or below the average national rate during the study period. Appendix B details how we calculated the case-mix–adjusted rates (AR) and the coefficient of variation (CV) between COROP regions (a measure of heterogeneity).

## Results

**Fig. 1:**
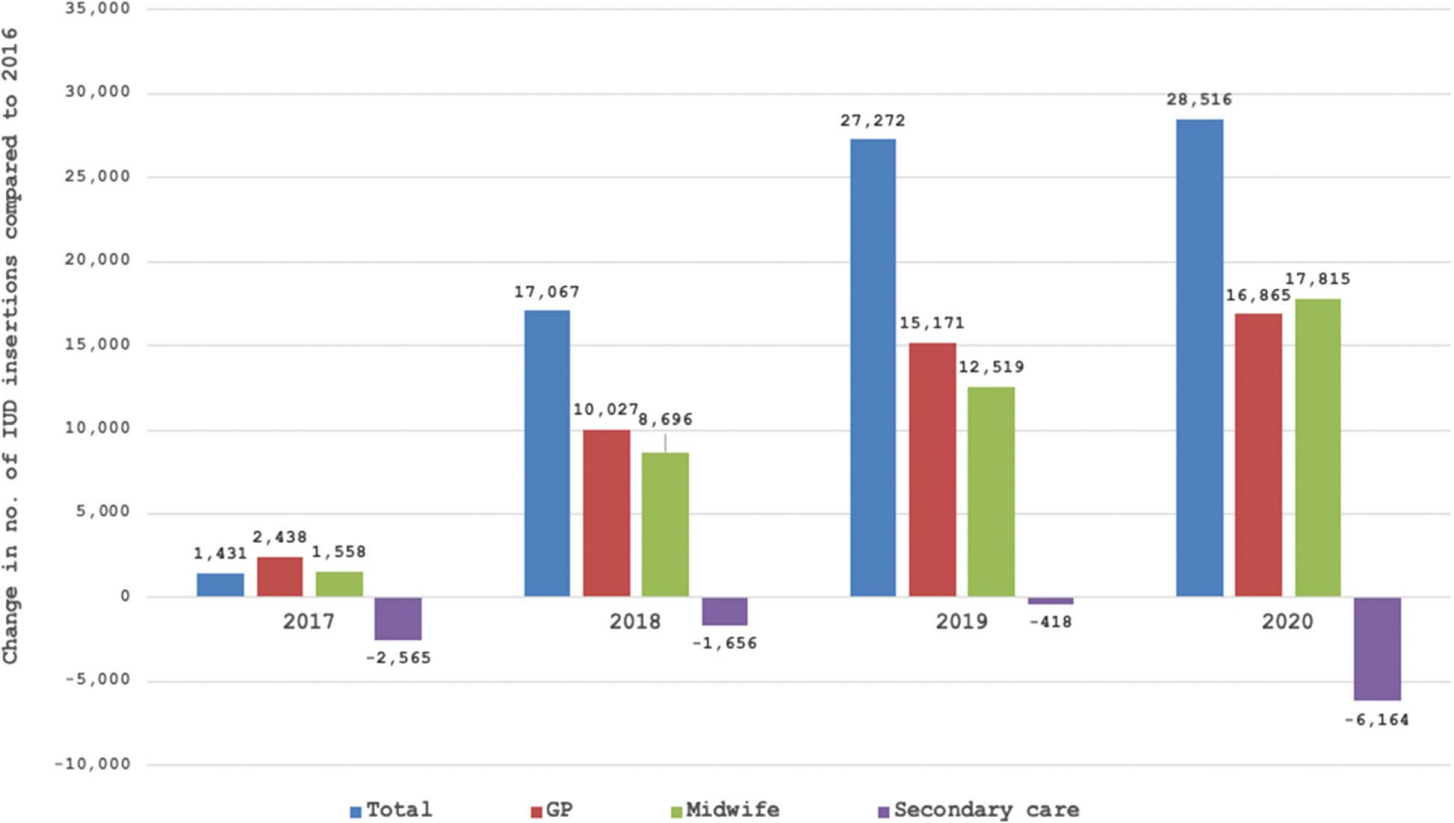
Change in number of IUDs per year compared to 2016, stratified by healthcare professional. Abbreviations: GP, general practitioner; IUD, intrauterine device

We studied 724,869 study participants who underwent 840,766 IUD insertions (Table 1). The crude ratios of IUD insertions for each type of health care professional are shown by year in Table 2. Between 2016 and 2020, GPs performed 2,978 IUD insertions per 100,000 person years (regional variance, 1,917–4,033; CV, 0.18), gynaecologists and residents performed 1,143 (regional variance, 778–1,561; CV, 0.16), and midwives performed 209 (regional variance,15–396; CV, 0.43). The total number of annual IUD insertions increased from 153,296 in 2016 to 181,812 in 2020. Figure 1 shows that half of this increase can be attributed to GPs and the other half to midwives.

**Table 1:** Baseline characteristics of study population (2016–2020)

**Characteristic**	
Women, n	724,869
Number of COROP regions, n	39
Mean number women per COROP region, n	20,644
Number of women with 1,2 or ≥ 2 IUDs within study period	
- *1, n (%)*	724,764 (86.0%)
- *2, n (%)*	101,912 (12.1%)
- > *2, n (%)*	16,269 (1.9%)
Practitioner	
- *inserted by GP, %*	68.8
- *inserted by midwives, %*	4.8
- *inserted by gynaecologists, %*	26.4
Number of ultrasounds	83,462
Number of IUD reinsertions	41,069
Patient age	
- ≤ *24 years, %*	23.7
- *25–34 years, %*	33.9
- *35–44 years, %*	27.4
- ≥ *45 years, %*	15.0
Ethnicity	
- *0%–20% non-western immigrants, %*	70.1
- *20%–40% non-western immigrants, %*	25.6
- > *40% non-western immigrants, %*	4.3
Household income	
- *low (p0–p40), %* - *moderate (p40–p80), %*	39.5 39.8
- *high (p80–p100), %*	20.7

*Abbreviations: COROP* (*Coördinatiecommissie Regionaal Onderzoeksprogramma*; literally, Coordination Commission Regional Research Program), *IUD* Intrauterine device

**Table 2:** Crude ratios of IUD insertions per type of health care professional

	2016–2020	2016^a^	2017	2018	2019	2020
**Total number IUDs**	840,766	153,296	154,727	170,363	180,568	181,812
**GP**						
n	578,211	106,742	109,180	116,769	121,913	123,607
Proportion inserted by GPs (regional variance)	69% (54%–81%)	70% (51%–81%)	71% (56%–81%)	69% (52%–83%)	68% (53%–79%)	68% (54%–82%)
Number per 100,000 person years	2,978	2,748	2,822	2,993	3,130	3,181
**Midwife**						
n	40,588	0	1,558	8,696	12,519	17,815
Proportion inserted by midwifes (regional variance)	5% (0%–10%)	0	1% (0%–4%)	5% (0%–11%)	7% (0%–14%)	10% (1%–19%)
Number per 100,000 person years	209	0	40	224	321	458
**Gynaecologist**						
n	221,967	46,554	43,989	44,898	46,136	40,390
Proportion inserted by the gynaecologist / resident (regional variance)	26% (18%–43%)	30% (19%–49%)	28% (19%–44%)	26% (16%–45%)	26% (18%–41%)	22% (16%–35%)
Number per 100,000 person years	1,143	1,199	1,137	1,157	1,185	1,039

^a^In the Netherlands, midwives have only been able to insert IUDs since 2017

*Abbreviations: GP* General practitioner, *IUD* Intrauterine device

**Fig. 2:**
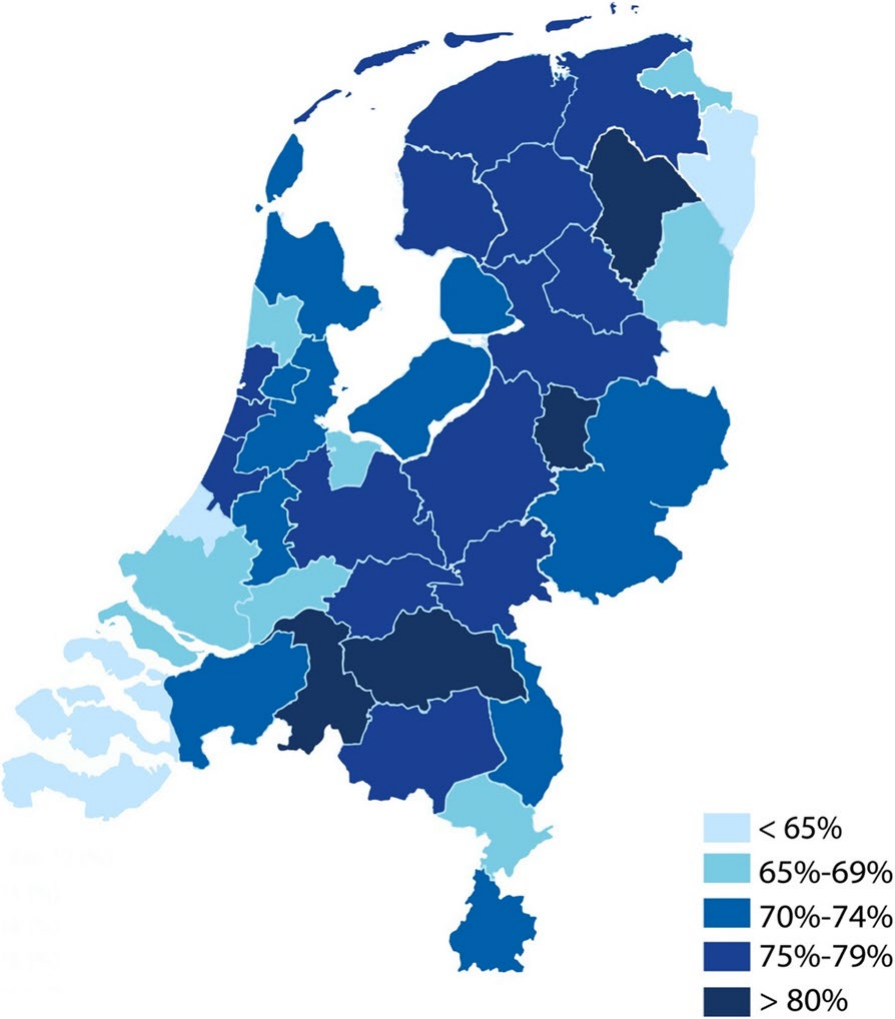
Case-mix–adjusted IUD insertion rate (2016–2020) in primary versus secondary care. The proportion of procedures in primary care is shown by intensity of blue. Abbreviations: IUD, intrauterine device

The results of the multilevel random effects logit models for the primary outcomes are shown in Table 3. While the case-mix–adjusted ratio for the overall percentage of IUDs placed in primary care was 74% (regional variance, 58%–82%; CV, 0.07) between 2016 and 2020, trend analysis revealed an increase in the ratio of IUDs inserted in primary care from 70% (regional variance, 53%–81%; CV, 0.10) in 2016 to 77% (regional variance, 65%–85%; CV, 0.06) in 2020. The *Oost-Groningen* region had the lowest case-mix–adjusted ratio every year (AR, from 53% in 2016 to 65% in 2020), whereas the region with the highest ratio changed from *Noord-Drenthe* in 2016 (AR, 81%) to *Zuidwest-Overijssel* in 2017 (AR, 81%), 2019 (AR, 83%), and 2020 (AR, 85%), via *Midden Noord Brabant* in 2018 (AR, 84%). Figure 2 shows the case-mix–adjusted ratios by COROP region for the entire study period, and Appendix C details those ratios for each year.

**Table 3:** Primary and secondary outcomes: results of the multilevel random effects logit models

Outcome	Adjusted rate	Crude rate	CV
***Primary outcome***			
*Case-mix–adjusted substitution rate (regional variance) by year*			
- 2016–2020	74% (58%–82%)	74% (57%-82%)	0.07
- 2016	70% (53%–81%)	70% (52%-81%)	0.10
- 2017	72% (56%–81%)	72% (55%-81%)	0.08
- 2018	74% (57%–84%)	74% (56%-84%)	0.07
- 2019	75% (59%–83%)	74% (59%-82%)	0.06
- 2020	77% (65%–85%)	78% (65%-85%)	0.06
***Secondary outcomes***			
*Case-mix–adjusted proportion of patients with the following:*			
- ultrasound after IUD insertion in primary care (regional variance)	3% (2%–5%)	3% (2%-5%)	0.21
- ultrasound after IUD insertion in secondary care (regional variance)	23% (17%–28%)	23% (18%-29%)	0.10
- IUD reinsertion in primary care (regional variance)	2% (1%–3%)	2% (1%-3%)	0.22
- IUD reinsertion in secondary care (regional variance)	6% (3%–8%)	6% (3%-8%)	0.16

*Abbreviations: GP* General practitioner, *IUD* Intrauterine device

**Fig. 3:**
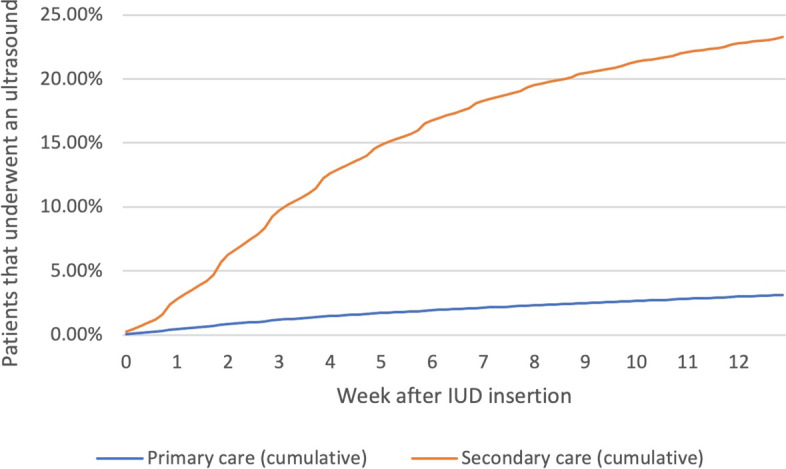
Percentage of patients who underwent ultrasound after IUD insertion. Abbreviations: IUD, intrauterine device

**Fig. 4:**
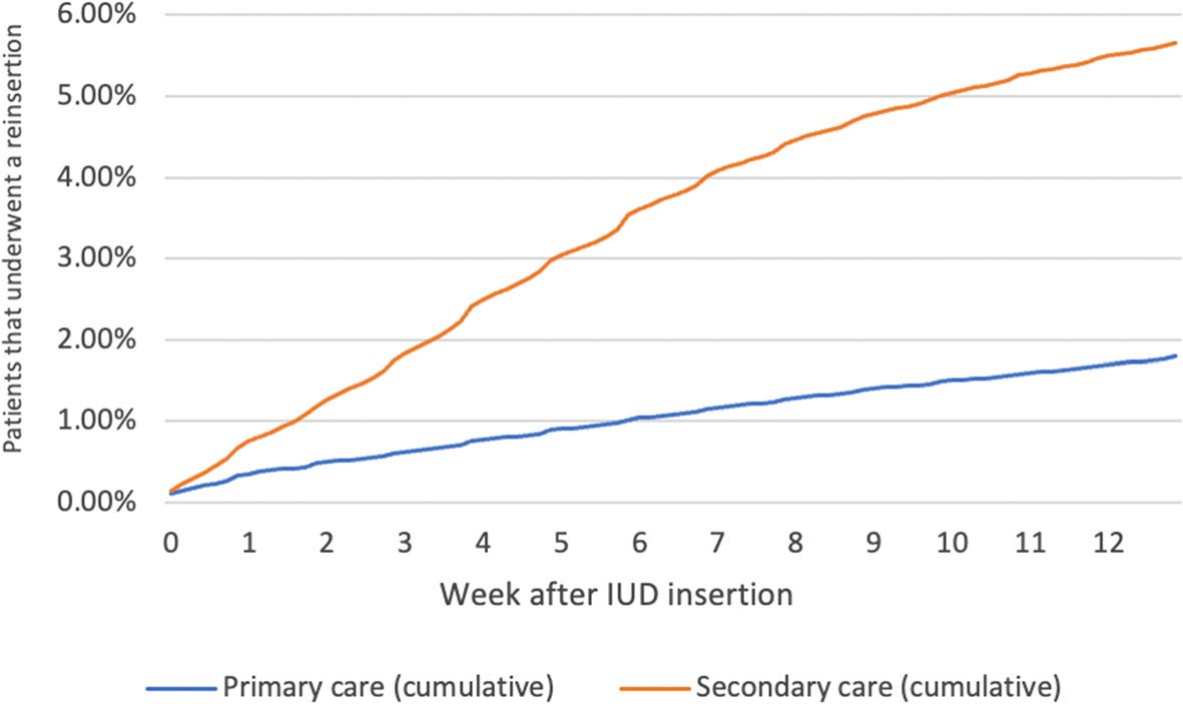
Percentage of patients who underwent reinsertion of the IUD. Abbreviations: IUD, intrauterine device

Table 3 also shows the results for the secondary outcomes. The overall case-mix–adjusted ratios for patients requiring ultrasound were 3% (regional variance, 2%–5%; CV, 0.21) in primary care vs. 23% (regional variance, 17%–28%; CV, 0.10) in secondary care (p < 0.01). Figure 3 shows the percentages of ultrasounds performed each week during follow-up. The overall percentage of patients requiring a new IUD insertion were 2% (regional variance, 0.01–0.03; CV, 0.22) in primary care vs. 6% (regional variance, 3%–8%; CV, 0.16) in secondary care (p < 0.01). Figure 4 shows the percentages and IUD reinsertions performed each week during follow-up.

Table 4 shows all outcomes by COROP region and the estimated results of each model.

**Table 4:** Outcomes by region

COROP region^a^	IUD / 100,000 person years			Adjusted substitution rate	Adjusted proportion ultrasound		Adjusted reinsertion rate	
	**GP**	**midwife**	**hospital**		**primary care**	**hospital care**	**primary care**	**hospital care**
Oost-Groningen	1,917	143	1,523	58%	3%	25%	3%	4%
Delfzijl en omgeving	2,387	15	1,153	69%	2%	24%	2%	4%
Overig Groningen	3,586	116	902	80%	2%	20%	1%	7%
Noord-Friesland	3,007	56	941	77%	2%	24%	2%	6%
Zuidwest-Friesland	3,000	105	976	77%	2%	28%	1%	6%
Zuidoost-Friesland	2,943	94	1,017	75%	2%	28%	2%	5%
Noord-Drenthe	3,226	100	849	80%	4%	25%	2%	8%
Zuidoost-Drenthe	3,124	138	1,561	68%	4%	26%	2%	4%
Zuidwest-Drenthe	3,149	102	861	80%	4%	27%	2%	8%
Noord-Overijssel	2,799	330	1,028	75%	3%	25%	1%	5%
Zuidwest-Overijssel	3,960	185	927	82%	2%	21%	2%	7%
Twente	2,659	360	1,149	73%	3%	23%	1%	4%
Veluwe	2,845	217	900	77%	3%	23%	2%	7%
Achterhoek	2,723	220	1,196	72%	2%	17%	1%	5%
Arnhem/Nijmegen	3,739	165	1,013	79%	3%	23%	1%	6%
Zuidwest-Gelderland	2,635	195	848	77%	3%	24%	2%	7%
Utrecht	3,579	170	1,037	78%	3%	25%	1%	5%
Kop van Noord-Holland	2,856	161	1,034	74%	4%	26%	2%	6%
Alkmaar en omgeving	2,626	308	1,283	69%	3%	25%	1%	5%
Ijmond	3,313	105	943	78%	4%	24%	2%	6%
Agglomeratie Haarlem	3,175	395	1,000	78%	4%	24%	2%	8%
Zaanstreek	2,810	278	1,238	72%	4%	20%	2%	6%
Groot-Amsterdam	3,109	239	1,312	71%	3%	24%	2%	6%
Het Gooi en Vechtstreek	2,962	98	1,405	69%	3%	24%	2%	5%
Agglomeratie Leiden en Bollenstreek	2,463	221	778	77%	3%	20%	2%	5%
Agglomeratie’s-Gravenhage	2,258	135	1,388	64%	4%	25%	2%	6%
Delft en Westland	2,880	103	1,381	68%	3%	27%	1%	6%
Oost-Zuid-Holland	2,488	217	1,045	72%	4%	24%	2%	5%
Groot-Rijnmond	2,403	224	1,332	67%	4%	20%	2%	5%
Zuidoost-Zuid-Holland	2,328	396	1,345	68%	3%	23%	1%	5%
Zeeland	2,138	69	1,253	64%	3%	23%	2%	6%
West-Noord-Brabant	3,254	155	1,211	74%	4%	21%	2%	6%
Midden-Noord-Brabant	4,033	43	925	81%	4%	27%	3%	7%
Noordoost-Noord-Brabant	3,826	200	992	80%	4%	21%	2%	8%
Zuidoost-Noord-Brabant	3,588	340	1,225	76%	5%	26%	2%	6%
Noord-Limburg	2,562	296	1,149	72%	4%	23%	1%	5%
Midden-Limburg	2,452	382	1,528	65%	4%	23%	2%	3%
Zuid-Limburg	2,861	347	1,119	74%	4%	23%	2%	5%
Flevoland	2,521	191	1,124	71%	3%	22%	2%	6%

^a^COROP: *Coördinatiecommissie Regionaal Onderzoeksprogramma*; literally, Coordination Commission Regional Research Program

*Abbreviations: GP* General practitioner, *IUD* Intrauterine device

## Discussion

The case-mix–adjusted percentage of IUD insertions in Dutch primary care increased from 70% in 2016 to 77% in 2020, showing variation of 58%–82% by COROP region. Given that the highest regional substitution rate was relatively stable throughout (i.e., 81% and 85%), we can suggest a realistic target of 80% for all regions. Professional role substitution by midwives could play a key role in achieving this target based on their growing contribution since receiving approval to perform the insertions in 2017, reaching 9% by 2020, albeit with variation of 1%–19% by COROP region. Encouraging midwives in regions with low uptake should improve this variation. Indeed, it was notable that midwives accounted for approximately half of the growth in absolute volume of IUD insertions since 2017. This growth could reflect either supplier-induced demand or a change in women’s preference for contraception, indicating the need for further research into the reasons for the change in preference.

Our results demonstrated that in addition to GPs, who have been placing IUDs for many years, midwives have gained a significant share in the number of IUD insertions in primary care in the Netherlands. This can be explained by both demand-side factors (women’s preference) and supply-side factors (midwives). On the demand side, women may prefer IUD insertion by a healthcare provider with whom they have had a positive experience during a previous childbirth. On the supply side, we observe a declining birth rate in the Netherlands, which may have allowed midwifery practices more time to focus on other activities, such as IUD insertions [18].

Furthermore, our results confirmed that hospital physicians request more ultrasounds (23% vs. 3%) and more IUD reinsertions (6% vs. 2%) than GPs and midwives in the first 3 months after IUD insertion. Several factors could account for the higher follow-up and reinsertion rates in the first 3 months after insertion observed among hospital physicians. For example, hospitals may treat a different, more severe, patient population, and we cannot exclude the possibility of insufficient correction for case mix. This is supported by the finding that fewer reinsertions were performed in regions where primary care health care professionals refer relatively more patients to the hospital for an IUD insertion (e.g., *Oost-Groningen* and *Midden-Limburg*). It could also reflect overdiagnosis due to the treatment of conditions that would otherwise not cause symptoms or harm a woman during her lifetime [19]. The literature also states that IUDs should be located near the fundus, though no exact cut-off point has been established [20]. When an IUD is inserted in the hospital, potentially unnecessary ultrasounds may be performed that diagnose clinically irrelevant malpositions, resulting in more follow-up visits and reinsertions. However, our dataset did not contain statistics on differences in pregnancy rates between IUD insertion in primary and secondary care, let alone the impact of malposition. However, from literature it is known that IUDs can safely be inserted by health care professionals in primary care [8]. Similarly, hospital residents, who may be less experienced, might perform more ultrasounds than necessary to verify the correct positioning of the IUD post-insertion.

Our results revealed several regions that showed a stable substitution rate of over 80% between 2016–2020. However, the region Oost-Groningen did not achieve 65%. Comparing these regions in terms of organization of health care and attitudes of health care professionals and patients towards IUD insertion in primary care, could provide valuable insights that could help conservative regions to increase their substitution rate.

Previous studies on regional practice variation of IUD insertions, have reported on both variation by general practitioners, and trends in the percentage of IUDs that is inserted in primary vs. secondary care. Contrary to our findings, Pahle et al. observed in Norway between 2011 and 2014 a shift of IUD-insertions from primary care to the hospital setting [12]. This study also showed that GPs with a higher number of registered patients and female GPs were associated with performing more IUD insertions. In Ontario, Canada between 2000 – 2006 the same trend towards performing IUD insertions in hospital setting was observed [21]. Moreover, the results of our study correspond to literature that identified medical practice variation between regions [12]. However, to our best knowledge no study assessed practice variation in the substitution rate of IUD insertions, or the difference in re-insertion rates and follow-up using ultrasound in primary versus secondary care.

Although health care substitution is set to become an increasingly important tool to reduce health care costs, it will have serious consequences for daily clinical practice. First, the case-mix of patients will become more complex for all health care professionals. For the gynaecologist, the substitution of IUD insertion will result in the loss of a relatively simple consultation that might be used to compensate financially for the time spent with more complex cases. By contrast, a primary health care processional might need to spend more time on IUD insertion because they are less experienced and work in a less-specialized environment. Second, the volume of patients seen in primary care will increase, affecting the workload of health care professionals. This situation is compounded in many countries by shortages of GPs [22–24], which could potentially affect the feasibility of substitution. However, as shown in this study, increasing the role of midwives could be key to delivering effective care substitution, especially because this does did not appear to affect the quality of health care for IUD insertions [8].

A major strength of our study was the completeness of the data. Vektis provided claims data with national coverage, facilitating the analysis of all IUD insertions in the Netherlands over a 5-year period and eliminating potential selection bias. Despite this strength, our study has several limitations. First, when performing the case-mix corrections, we used socioeconomic status and ethnicity as proxies for obesity and uterine myomas, respectively. Although correlations exist between these variables, they do not capture the variables of interest exactly. Second, we were not able to address the safety of IUD insertions or whether the higher ultrasound and reinsertion rates in secondary care resulted in fewer unwanted pregnancies. Third, registration bias may affect our study if procedures such as IUD insertions (codes 13042, 1719, 037180, 190274) or ultrasounds (code 039492) are (unintentionally) not billed by healthcare providers to the health insurer. Finally, based on our data, it was not possible to distinguish which patients had a strict medical indication for undergoing intrauterine device IUD insertion in secondary care.

## Conclusions

The proportion of intrauterine devices (IUDs) inserted in primary care facilities as opposed to hospital care settings in the Netherlands witnessed a growth from 70% in 2016 to 77% in 2020. The distribution of these percentages exhibited variations across different regions, spanning from 58 to 82%. Therefore, we propose a rate of 80% as a realistic target for substitution, consistent with relatively stable peak substitution rates of 81%–85% among regions. Given that midwives not only contributed to about half of the increase in care substitution but also accounted for the largest regional variation, they could play a key role in achieving this target substitution rate. Finally, our findings suggest that moving the responsibility for IUD placement to primary care could reduce the number of ultrasounds and IUD reinsertions performed during 3-month follow-up after IUD insertion.

### Supplementary Information

Supplementary Material 1

Supplementary Material 2

Supplementary Material 3

## Data Availability

The data that support the findings of this study are available from Vektis but restrictions apply to the availability of these data, which were used under license for the current study, and so are not publicly available. Data are however available from the authors upon reasonable request and with permission of Vektis.

## Abbreviations

DRG: Diagnosis Related Groups
DTC: Diagnosis Treatment Combinations
GP: General practitioner
IUD: Intrauterine device
SES: Socioeconomic status
