# Supplementary material for: Regional variation in health care substitution for intrauterine device insertion: a retrospective cohort study
Source: BMC Prim Care. 2024 Aug 10;25:294. doi: 10.1186/s12875-024-02546-7 (PMC11316978; doi:10.1186/s12875-024-02546-7)
Supplement: Supplementary file 1 — Supplementary Material 1 [file 12875_2024_2546_MOESM1_ESM.docx]

Appendix A: Health care activity codes and specification of logit models

| **Health care activity code** | **Description** |
| --- | --- |
| S1-verrichtingscode 13042 | IUD placement general practitioner |
| Midwife code 1719 | IUD placement midwife |
| 037180 | IUD placement gynaecologist |
| 190274 | IUD product code (hospital setting) |
| 039492 | Ultrasound performed |

**Model 1:** Ratio of IUD insertion by general practitioners and midwives versus gynaecologists, reported at the national level and by COROP region.

*Numerator:*

- Number of IUD insertions by GPs and midwives, identified by health care activity code S1-verrichtingscode 13042 or midwife code 1719.
- Timeframe: January 1^st^ 2016 to January 1^st^ 2021;
  - For the whole period and separately reported for 2016, 2017, 2018, 2019, and 2020.

*Denominator:*

- Sum of IUDs with health care activity code 037180 or 190274 **OR** S1-verrichtingscode 13042 or midwife code 1719.
- Timeframe: January 1^st^ 2016 to January 1^st^ 2021;
  - Separately reported for 2016, 2017, 2018, 2019, and 2020.

**Model 2:** Ratio of ultrasounds after IUD insertion by general practitioners and midwives, reported at the national level and by COROP region.

*Numerator:*

- Sum of ultrasounds: Health care activity code 039492 registered within 3 months after IUD insertion by GP or midwife.
- Timeframe: January 1^st^ 2016 to January 1^st^ 2021.

*Denominator:*

- Sum of IUD insertions by the general practitioner and by the midwife, identified by S1-verrichtingscode 13042 or midwife code 1719.

**Model 3:** Ratio of ultrasounds after IUD insertion by gynaecologists, reported at the national level and by COROP region.

*Numerator:*

- Sum of ultrasounds: Health care activity code 039492 registered within 3 months after IUD insertion by a gynaecologist, but not on the day of IUD insertion.^[[1]](#footnote-1)^
- Timeframe: January 1^st^ 2016 to January 1^st^ 2021.

*Denominator:*

- Sum of IUD insertions by gynaecologists, identified by health care activity code 037180 or 190274.

**Model 4:** Ratio of IUD replacements (new IUD insertions) after initial IUD insertion by general practitioners and midwives, reported at the national level and by COROP region.

*Numerator:*

- Sum of replacements: health care activity code 037180 or 190274 **OR** health care activity code S1-verrichtingscode 13042 or midwife code 1719 registered within 3 months after initial IUD insertion by GP or midwife.
- Timeframe: January 1^st^ 2016 to January 1^st^ 2021.

*Denominator:*

- Sum of IUD insertions by general practitioners and midwives, identified by S1-verrichtingscode 13042 or midwife code 1719.

**Model 5:** Ratio of IUD replacements (new IUD insertion) after initial IUD insertion by gynaecologists, reported at the national level and by COROP region.

*Numerator:*

- Sum of replacements: health care activity code 037180 or 190274 **OR** S1-verrichtingscode 13042 or midwife code 1719 registered within 3 months after prior IUD placement by a gynaecologist.
- Timeframe: January 1^st^ 2016 to January 1^st^ 2021.

*Denominator:*

- Sum of IUD insertions by gynaecologists, identified by health care activity code 037180 OR 190274.

**Case-mix correction**

All models underwent case-mix correction for the following factors:

- Age;
- Household income (proxy for socioeconomic status);
- Percentage of non-Western immigrants (proxy for ethnicity).

1. The gynecologist usually performs an ultrasound directly after IUD placement. As we are interested in the number of additional follow-up visits, only ultrasounds at least one day after IUD placement were considered. [↑](#footnote-ref-1)
