# Supplementary material for: Regional variation in health care substitution for intrauterine device insertion: a retrospective cohort study
Source: BMC Prim Care. 2024 Aug 10;25:294. doi: 10.1186/s12875-024-02546-7 (PMC11316978; doi:10.1186/s12875-024-02546-7)
Supplement: Supplementary file 2 — Supplementary Material 2 [file 12875_2024_2546_MOESM2_ESM.docx]

Appendix B

**Case-mix adjustment**

The case-mix–adjusted rates (AR) were computed as follows:

$$I_{j}^{k}=\left( Y_{j}^{k}-E(Y_{j}^{k}) \right)+ Y^{k}$$

where I denotes the case-mix–adjusted outcome measure k (k = 1–5), j refers to the COROP region, $Y_{j}^{k}$ is the crude outcome region j, $E(Y_{j}^{k})$ is the expected outcome k based on the case-mix characteristics of the region j and $Y^{k}$ refers to the population mean of crude outcome k.

**Measurement of heterogeneity**

To estimate the extent of the regional practice variation in the substitution of IUD insertions, we calculated the coefficient of variation (CV) between COROP regions as a descriptive measure of heterogeneity. This was calculated by dividing the standard deviation by the mean of the COROP-specific random parameters.
