# Supplementary figures and images for: Regional variation in health care substitution for intrauterine device insertion: a retrospective cohort study
Source: BMC Prim Care. 2024 Aug 10;25:294. doi: 10.1186/s12875-024-02546-7 (PMC11316978; doi:10.1186/s12875-024-02546-7)

**
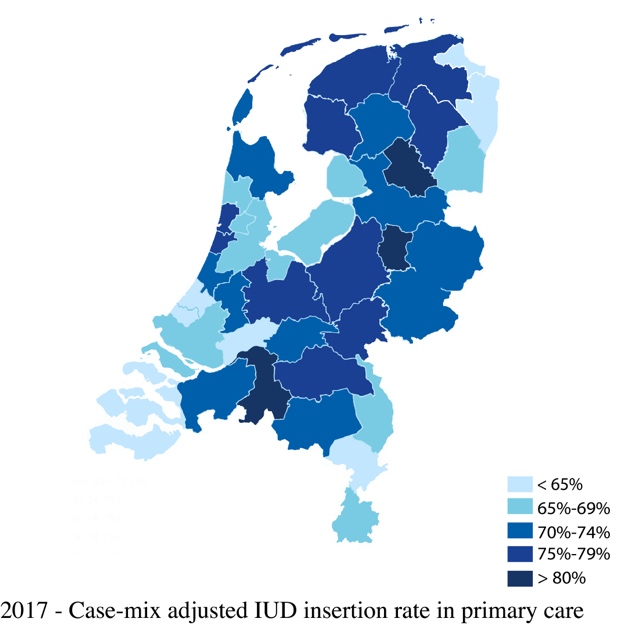

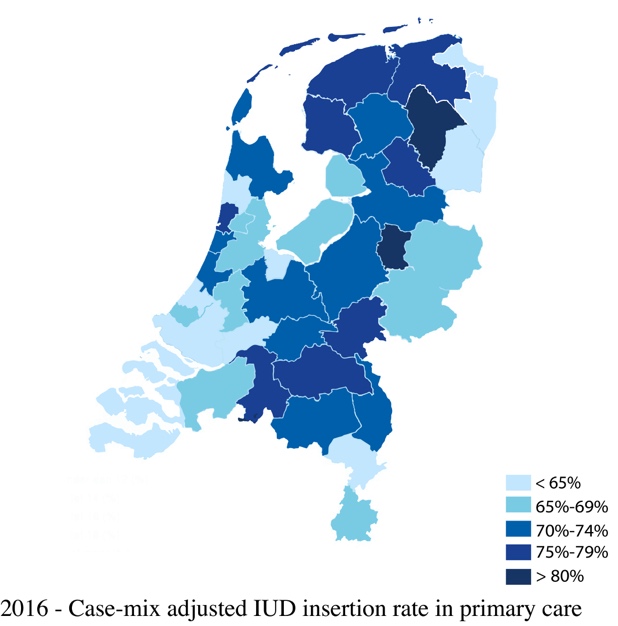
**Appendix C: Regional practice variation of substitution rates per year


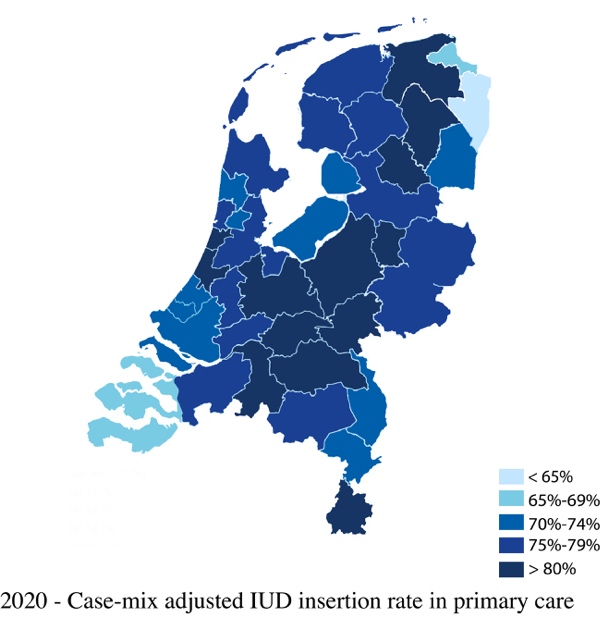
**
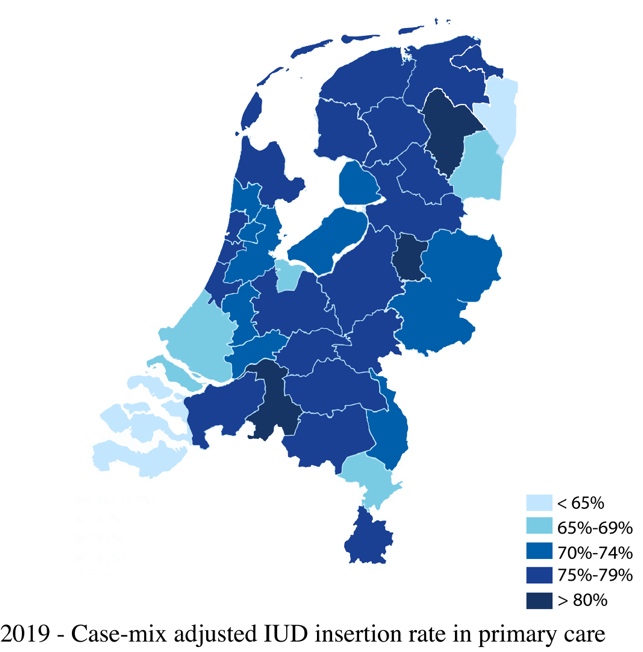

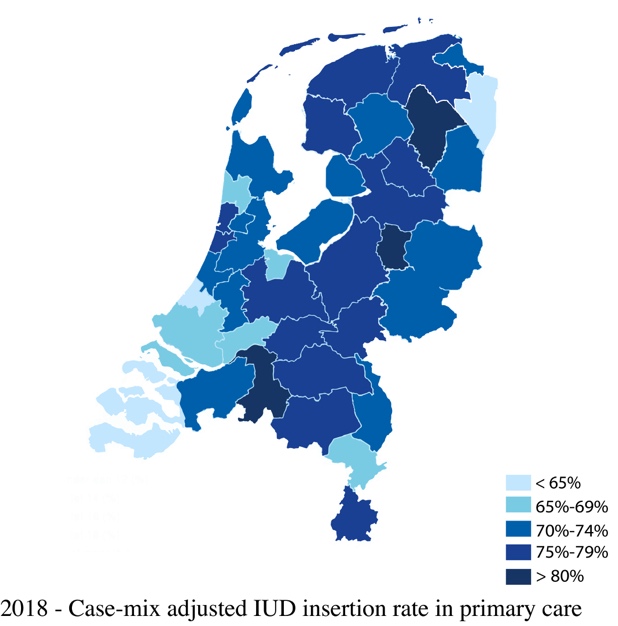
**

Supplement: Supplementary file 3 — Supplementary Material 3 [file 12875_2024_2546_MOESM3_ESM.docx]
